# Physical activity counseling in maternity and child health care – a controlled trial

**DOI:** 10.1186/1472-6874-8-14

**Published:** 2008-08-14

**Authors:** Minna Aittasalo, Matti Pasanen, Mikael Fogelholm, Tarja I Kinnunen, Katriina Ojala, Riitta Luoto

**Affiliations:** 1 The UKK Institute for Health Promotion Research, Tampere, Finland; 2 Health Research Unit, Academy of Finland, Helsinki, Finland; 3 Tampere School of Public Health, University of Tampere, Tampere, Finland

## Abstract

**Background:**

The purpose of the study is to examine the effects and feasibility of individual physical activity (PA) counseling in maternity and child health clinics in Finland.

**Methods:**

Three clinics including both maternity and child health care signed up for the experimental (EXP) and three for the control group (CON). The participants were 132 pregnant and 92 postpartum primiparas. The nurses in EXP integrated a primary and four booster PA counseling sessions into routine visits. An option for supervised group exercise was offered. In CON former practices, usually including brief PA advice, were continued. Leisure-time PA (LTPA) prior to pregnancy was elicited by questionnaire and followed 16–18 and 36–37 weeks' gestation in maternity clinics and 5 and 10 months postpartum in child health clinics. Feasibility included safety, participant responsiveness, realization of counseling and applicability.

**Results:**

According to analysis of covariance adjusted for baseline LTPA and possible confounders, no relative between-group differences in LTPA were found at the first follow-up in either maternity or child health clinics. At the last follow-up in maternity clinics the weekly number of at least moderate-intensity LTPA days was 43% (95% CI: 9, 87) higher and the weekly duration of at least moderate-intensity LTPA 154% (95% CI: 16, 455) higher in EXP compared with CON. Counseling proved feasible in both maternity and child health clinics.

**Conclusion:**

Counseling encouraged pregnant women to sustain their moderate-intensity LTPA and was feasible in routine practices. No effects were observed if counseling was initiated postpartum.

**Trial registration:**

Current Controlled Trials ISRCTN21512277

## Background

The health benefits of physical activity (PA) among general population are well documented [1,2]. Nevertheless, only less than half of the population in the developed countries is sufficiently physically active [2,3]. Women, in general, seem to be more inactive than men, especially in moderate-to-vigorous-intensity PA [4].

Women's PA may decrease further during pregnancy [5-7] due, for example, to physical limitations [6] or due to fear of harming the fetus [6,8]. Similar unfavorable changes in PA have also been discovered during postpartum [6,9] mainly due to responsibilities related to childcare [6,10]. Pregnant and postpartum women also tend to shift from moderate to light-intensity PA [11,12]. However, according to the CDC-ACSM recommendation [2], which is also valid during uncomplicated pregnancy and postpartum period [13-15], to achieve health effects, accumulation of moderate-intensity PA on most days of the week is needed.

PA should, thus, be highlighted by health care professionals both during uncomplicated pregnancy [14,16] and postpartum [17]. This is further supported by the fact that PA prior to, during, and after pregnancy is positively associated with overall health status [18]. More specifically, it can prevent gestational diabetes, especially among obese women [19], decrease postpartum weight retention [20] and promote postpartum psychological well-being [8,9]. Women may also be more susceptible to behavioral modifications during pregnancy [14,16] and postpartum [21].

Currently, PA counseling is not, however, routinely integrated into pre- and postpartum care [8,9]. Also, the counseling is unsystematic [22], which may partly be due to insufficient evidence on the best way to implement counseling [16]. In most of the studies the effects of instructed exercise, not the effects of individual counseling are examined. Furthermore, the aim of the studies has most often been to prevent excessive weight gain and thus, the total energy expenditure or overall PA has been used as an outcome. In the study by Polley et al. [23] education and behavioral strategies were used in order to achieve the recommended rates of pregnancy weight gain. As a result, no changes in energy expenditure were discovered. Similar findings have been reported by Gray-Donald et al. [24] among Cree pregnant women. Regarding postpartum women, O'Toole et al. [25] found that an educational intervention was effective in increasing subjects' energy expenditure over a period of one year but the high drop-out rate of 43% impairs the value of the results. Also, in the earlier publications related to this present study it was discovered that individual counseling on PA and diet did not increase total leisure time energy expenditure among pregnant or postpartum women [26,27].

However, no studies have been published about the effects of counseling on the PA patterns of pregnant and postpartum women. The purpose of this study is 1) to examine the effects of individual counseling on the frequency and duration of light and at least moderate-intensity leisure-time PA (LTPA) among pregnant and postpartum women and 2) to evaluate the feasibility of the counseling procedure implemented in real health care setting.

## Methods

### Participants

**Figure 1:**
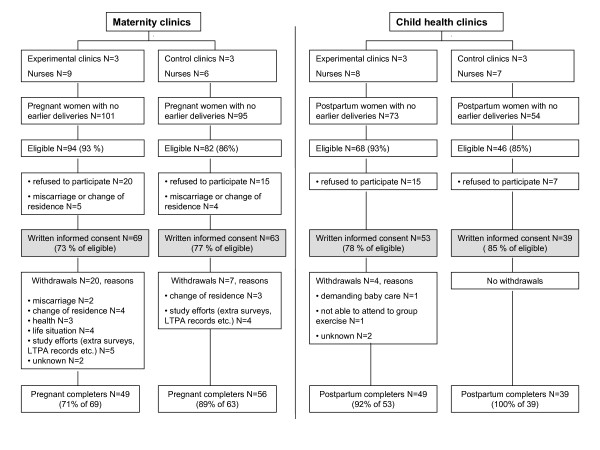
Participant flow in the maternity and child health clinics.

The study was approved by the ethical committee of the Pirkanmaa Hospital District. A convenience sample of six municipal clinics, each including both maternity and child health care, from two southern cities participated in the study. Three of the clinics volunteered for the experimental group (EXP) and the rest were assigned to the control group (CON) (Fig 1). From the clinics altogether 24 public health nurses, referred here as nurses, participated in the study. Of these, nine worked in maternity clinics (MCs), eight in child health clinics (CCs) and seven at both clinics.

The nurses recruited women with no earlier deliveries in most cases by telephone each time a women set the appointment time for the first visit (in MCs 8–9 weeks' gestation and in CCs 2 months after delivery). The exclusion criteria were age under 18 years, type 1 or 2 diabetes mellitus, twin pregnancy, otherwise problematic pregnancy (e.g. increased risk for miscarriage), physical disability, substance abuse, treatment or clinical history of any psychiatric illness, inability to speak Finnish and intention to change place of residence within the next three months. Preliminarily eligible women were mailed a baseline questionnaire with a request to return in on the first visit, where eligibility was confirmed and written informed consent obtained.

### Physical activity counseling

#### Experimental clinics

**Figure 2:**
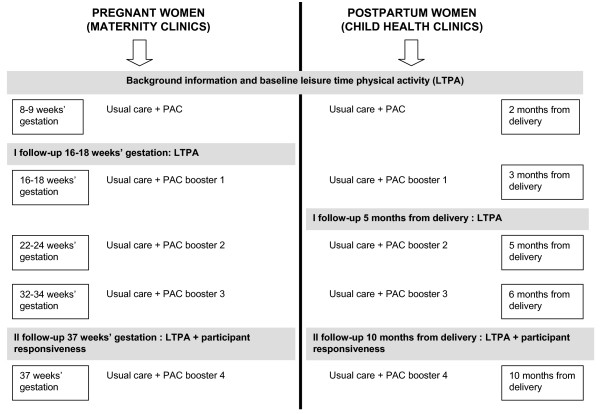
Timing of physical activity counseling (PAC) and data collection in the experimental clinics.

In Finland, nurses are recommended to see their primiparous clients 11–15 times during pregnancy [28] and 10 times during the child's first year [29]. The services are provided free of charge. One primary and four booster PA counseling sessions were integrated into five of the routine visits (Fig. 2). An option for supervised group exercise (60') once a week arranged close to the clinics was offered to provide supportive relationships for behavior change, which has been shown to be important in earlier studies [6]. The possibility was presented to the participants by the nurses at the first visit and reminded in the course of the study by the nurses and one of the researchers. The group exercise consisted of three types of training: walking, low impact aerobic and circuit training. In the postpartum group, the babies were involved in the training.

The structure and the topics of counseling follow the model introduced by Laitakari and Asikainen [30]. The sessions were guided by the counseling card, particular for each participant and including the topics with writing space for each session. The topics during the primary session were: LTPA prior to pregnancy, current LTPA, need for increasing LTPA, current guidelines for PA during pregnancy with the help of a take-home leaflet, barriers and incentives to LTPA. At the end of discussion, a weekly plan including LTPA modes and their frequency, duration and intensity was made. Before the intervention, the nurses had a one-day training session, an obligation to practice counseling with one of person not participating in the study and a two-hour feedback session in small groups where it was possible to more individually assess the nurses' counseling skills. The stages of change [31] were introduced to the nurses in the training and they were instructed to consider each participant's readiness to increase LTPA and to proceed within these limits. During the intervention, one supportive meeting was held and monthly researcher visits were made to the clinics.

In making the action plan Borg's [32] visual scale of perceived exertion (RPE) including ratings 6–20 was used to help the participants to assess the intensity of each activity mode. The focus was on promoting LTPA with RPE ratings 12–14 (somewhat hard) as suggested in the current guidelines on exercise during pregnancy and postpartum [13-15]. It was estimated by the researchers from the CDC-ACSM recommendation [2], which can be applied during uncomplicated pregnancy and postpartum period [13-15], that the minimum LTPA required for fitness was 840 MET minutes per week (40 minutes of moderate to high-intensity PA [7 METs] 3 times per week) and for health 750 MET minutes per week (30 minutes of moderate-intensity PA [5 METs] on 5 weekdays). Both aspects, health and fitness, were considered because some women may have preferred to continue with their more high-intensity LTPA. Thus, a consensus of 800 MET minutes was taken to be the minimum dose of LTPA gradually included in the plan. In the updated PA recommendation for the general adult population, which was published in after the intervention, the minimum goal for health was clarified as 750 met minutes of moderate-intensity PA [33] suggesting that our estimation was quite appropriate.

The nurses calculated the MET minutes from the plan by multiplying the weekly minutes and MET value of each LTPA mode and summing the figures. Concluded from [15,34] RPE 6–11 equaled three METs (light), 12–14 five METs (moderate) and 14–20 seven METs (high). The MET minute criterion was set to show the nurse whether there were still need to encourage the participant to increase her weekly LTPA at the next visit; it was not discussed with the participants.

Compliance with the plan was assessed at the booster sessions (in MCs 16–18, 22–24, 32–34 and 37 weeks' gestation and in CCs 3, 5, 6 and 10 months after delivery) with the help of a LTPA log attached to the plan. If the plan was complemented or revised, the MET minutes were rechecked. Other topics at the booster sessions were: adverse events after previous visit, adverse events related to LTPA, barriers and incentives to comply with the plan. The time allocated for the primary counseling was 20–30 minutes and for each of the boosters 10–15 minutes.

#### Control clinics

Former PA counseling practices were continued in CON. According to the responses (N = 22) to a questionnaire delivered to all the participating nurses at baseline, 13 of the 14 nurses in MCs discussed PA at each woman's first visit. The mean duration of discussion was 7.5 minutes. In CCs, only 5 of the 15 nurses discussed PA at each woman's first visit with a mean duration of 3.9 minutes. The topics raised most frequently were pregnancy related physiological changes and present PA habits. There were no differences between EXP and CON in the number of nurses implementing PA counseling or mean durations (minutes) of PA discussions at the first visit in MCs (range 4–13 vs. 3–10) or CCs (range 0–8 vs. 0–8). Thus, regardless of voluntary sampling, the practices in CON seemed to represent the former counseling practices of this sample. Regarding the study arrangements and data collection one training and one feedback session was arranged for the nurses before the intervention and researcher visits were made as to the experimental clinics.

### Evaluation of effects

A baseline and two follow-up LTPA questionnaires were completed in MCs and CCs to compare the changes in weekly at least moderate-intensity and light-intensity LTPA between EXP and CON (Fig. 2). The baseline questionnaire elicited LTPA during a typical week prior to the pregnancy and the follow-up questionnaires elicited LTPA during a typical week during the previous three weeks. The questions were modified from the International Physical Activity Questionnaire (IPAQ) eliciting the frequency and duration in minutes of vigorous, moderate and light-intensity PA [35]. Contrary to the original IPAQ, the intensities were expressed as degree of breathlessness (strong, some, none) because there are indications that for some women, the meaning of intensity may be difficult to understand [36]. For example, the women were first asked on how many days per week they were engaged in LTPA which causes strong breathlessness (weekly number of days with vigorous-intensity LTPA). Then, the average duration of one session with this kind of intensity was elicited. The weekly duration of vigorous-intensity LTPA was calculated by multiplying the number of days by average duration of one session. Similar procedure was followed with other intensities. To calculate the duration of at least moderate-intensity LTPA, the weekly durations of vigorous and moderate-intensity LTPA were summed up.

### Evaluation of feasibility

The feasibility of the whole study has been reported elsewhere [37]. The feasibility of PA counseling was evaluated with four components: i) safety, ii) participant responsiveness, iii) realization of counseling and iv) applicability. The first two were compared between EXP and CON, the latter two concerned only EXP. The indicators and evaluation methods regarding each of the components are described in Additional file 1.

### Statistical methods

Descriptive information is given as arithmetic means, standard deviations (SD) and percentages. Group differences regarding each LTPA outcome variable adjusted for corresponding LTPA value at baseline and selected confounders were examined by analysis of covariance at the end point of both follow-ups. Each possible confounder was removed one by one from the analysis to examine, which of them had impact on the results. Thus, the selection was not dependent on their statistical impact. Before analysis, the transformations to logarithms of original variables were performed due to skewness of distributions regarding light-intensity LTPA. The results at the end point of each follow-up are illustrated as relative group differences (%) calculated using antilogs of mean differences of log-transformed variables and given with 95% confidence intervals. Analysis of covariance with the same adjustments was also used in feasibility evaluation by comparing the means between EXP and CON in counseling satisfaction (Osgood scale, 1–5), birth weight and weeks' gestation at delivery. The group difference in the perceived effects of counseling (6 categories) was examined with Chi square test and in the incidence of each selected adverse event with Chi square or Fisher's exact test. After dropping out, no follow-up information was collected from the participants.

## Results

The nurses recruited altogether 132 pregnant and 92 postpartum women during a period of six months (Fig. 1). Baseline information on participants is presented in Additional file 2. Pregnant women in EXP had higher BMI (t-test, p = 0.030) and had less often high education (Chi square, p = 0.009) than in CON. Among postpartum participants there were no statistical baseline differences between the groups.

The pregnant women withdrawing (N = 27) did not differ statistically from the ones completing the study (N = 105). BMI could not be compared because only the maternity cards of the completers were copied. Postpartum non-completers (N = 4) had significantly higher BMI (t-test, p = 0.029) than the completers (N = 88). Participants' LTPA at baseline and at both follow-ups in EXP and CON are presented as unadjusted arithmetic means in Additional file 3.

Among pregnant participants at the first follow-up there were no differences in LTPA between EXP and CON (Additional file 3). At the second follow-up the weekly number of at least moderate-intensity LTPA days was 43% (95% CI 9 to 87) higher and the weekly duration of at least moderate-intensity LTPA 154% (95% CI 16 to 455) higher in EXP compared with CON. Conversely, the weekly number of light-intensity LTPA days was 24% (95% CI 41 to 3) lower in EXP than in CON. Among postpartum participants no group differences in LTPA were discovered at either of the follow-ups.

There were no statistically significant differences between EXP and CON in the incidence (%) of selected adverse events among pregnant and postpartum participants. Among pregnant participants, one of the two miscarriages in EXP was due to blighted ovum and the other due to unknown causes before the first booster session. Among pregnant completers the mean birth weight of children was 3401 g (SD 341) in EXP and 3440 g (490) in CON and the adjusted mean between-group difference -44 g (95% CI -241 to 153). The mean weeks' gestation at delivery was 39.9 in both EXP (SD 1.45) and CON (SD 1.28) and the adjusted mean between-group difference 0.2 weeks (95% CI -0.4 to 0.8).

The score for counseling satisfaction was on average 3.7 (SD 0.8) in EXP and 2.9 (1.0) in CON among pregnant participants with a mean between-group difference of 0.7 (95% CI 0.3 to 1.1). Among postpartum participants the corresponding means were 3.3 (0.9) in EXP and 2.8 (0.7) in CON with a mean difference of 0.5 (95% CI 0.1 to 0.9) between the groups. Pregnant participants in EXP reported more often than their peers in CON that counseling had been an incentive to try out, initiate or maintain LTPA (81% vs. 43%, *p *< 0.001). A similar finding was made among postpartum participants (70% vs. 30%, *p *= 0.012).

The timing of the counseling sessions was as intended in both MCs and CCs. In MCs, the mean length was 25.6 (SD 8.1) minutes for the primary session and 12.1 (5.6) minutes for the boosters. In CCs, the corresponding means were 28.3 (11.1) and 11.9 (5.6) minutes. Five boosters were missed both in MCs and CCs. Adherence to group exercise was 28% among pregnant and 47% among postpartum participants. The nurses scored (1–5) the applicability of the primary counseling session to the routine visits 3.9 (SD 0.6) in MCs and 3.8 (0.4) in CCs.

## Discussion

Individual PA counseling supported with an option for supervised group exercise seemed to encourage pregnant women to sustain their pre-pregnant level of at least moderate-intensity LTPA almost until the end of their pregnancies. Among postpartum women, no statistically significant effects were observed. The realization and participant responsiveness of the counseling procedure were high and it seemed to be safe and applicable to routine care.

### Effects

Regarding pregnant women the findings seem encouraging and quite rational: During the first and second trimesters, when the physical restrictions due to pregnancy are still small, the level of pre-pregnancy LTPA can be maintained without extra support. However, as the pregnancy proceeds, PA counseling seemed to encourage the participants in EXP to continue with their more intensive LTPA while in CON, a tendency to shift towards more light-intensity LTPA can be observed. As a result, a statistically significant between-group difference can be seen at the last follow-up in the number of days of light-intensity LTPA in favor of CON.

The findings are in line with earlier reports about the change in LTPA patterns during pregnancy [6,11,12]. It seems, thus, that the sustainability of at least moderate-intensity LTPA during pregnancy may be supported by individual PA counseling. This is encouraging from the health promotion point of view, especially since it has been shown that women who integrate exercise into their routine during pregnancy continue exercising after delivery [38,39].

Among postpartum women there were no similar findings. The most reasonable explanation may be that, as the baseline LTPA was already high, the EXP participants' capacity to increase it may have been limited especially due to new child care responsibilities. Thus, counseling was not able to overcome these barriers. This is supported in earlier studies showing that lack of time due to family duties is the major barrier for PA after delivery [39,40]. However, a positive finding, also observed by others [41], was that pre-pregnancy LTPA was resumed shortly after delivery.

### Feasibility

The counseling supported by an optional group exercise appeared to be a *safe *way to promote PA among pregnant and postpartum women since no higher incidences of adverse events were reported in EXP than CON, and since the birth weights of the children and weeks' gestation at delivery were similar among pregnant participants' in both groups. The better scores in EXP on *participant responsiveness *may indicate a need for more intensive PA counseling in maternity and child health care. The *realization *of the counseling procedure was good, which strengthens the validity of the effects. The exception was pregnant women's poor adherence to the optional group exercise, which may suggest that they experience less need for peer support than postpartum women. Working schedules, tiredness after work and no perceived need for maternity exercise at the beginning of pregnancy may be speculated other reasons for less interest. The *applicability *score was high considering the time spent on PA discussions at baseline. This may reflect the flexibility of organizing the contents of the visits.

### Strengths and limitations

The strengths of the study were that 1) the counseling was based on a behaviorally grounded model, which had been found applicable in a health care setting, 2) the nurses were carefully trained for counseling, practiced counseling before the intervention and were supported in the implementation throughout the study and 3) a feasibility evaluation was included. Also, the probability of cross-over effect was minimal because neither the participants nor the nurses moved from EXP to CON or vice versa.

However, there were some limitations in the study. First, the sample was quite small, which may have weakened the statistical power to discover small between-group differences. Second, due to voluntary sampling of the clinics, the nurses in EXP may have had more favorable attitudes towards counseling than their peers in CON, leading to stronger counseling effects. On the other hand, the counseling practices on PA at baseline were quite similar in EXP and CON indicating no major differences in the attitudes.

Third, despite the representativeness of the women recruited – practically all pregnant [42] and postpartum women [29] use the municipal maternity and child health care services in Finland – the recruitment itself may have led to a selection bias and to the participation of the most compliant women in EXP, which is a common problem in intervention studies. However, at baseline, there were more pregnant and postpartum participants with BMI below 25 in CON than in EXP. Also, the number of pregnant participants with higher-level education was higher in CON than in EXP. As lower BMI [7,43,44] and higher level of education [5,44,45] have both been shown to be positively associated with women's PA, the participants in CON may have looked more favorably on LTPA than the participants in EXP. On the other hand, as the study proceeded, there were more withdrawals in EXP compared to CON and among pregnant women the dropouts seemed more likely to be non-exercisers than the completers.

Fourth, the LTPA questionnaire had not been validated for pregnant and postpartum women. A questionnaire for pregnant women was introduced only after the initiation of the study [46]. The sensitivity of the questionnaire used in this intervention may have been insufficient to discover the changes especially in light-intensity LTPA, which has also been recognized in other studies on women's PA [47,48]. It is also possible that women in EXP, being aware of expectations, were more likely than women in CON to over-report their LTPA at the follow-ups. The use of RPE may have improved the ability of the women in EXP to classify the intensity of their LTPA affecting to their responses in the follow-up questionnaires. There may also have been severe recall errors in the assessment of baseline LTPA due to retrospectivity of the questionnaire (approximately 2 months in the pregnant and 10 months in the postpartum group). However, the possible errors were expected to be the same in EXP and CON and should not have affected to group comparisons. Finally, the results may only be generalized in the clinics, which are interested in developing their counseling practices.

## Conclusion

General recommendations on PA are valid also during uncomplicated pregnancy and postpartum period. However, women tend to shift from health enhancing moderate-intensity activities to light-intensity activities during pregnancy. In this study, individual PA counseling supported with optional group exercise seemed to encourage pregnant women to sustain better their pre-pregnant level of at least moderate-intensity LTPA. No benefits for LTPA were observed if counseling was initiated postpartum. Counseling proved safe and applicable to routine practices and was more rewarding to pregnant and postpartum women than usual counseling. Adherence to group exercise was higher among postpartum than pregnant women. However, the limited sample size and unvalidated measures prevent making direct conclusions. Studies with larger sample sizes and validity-tested measures are therefore warranted. Also, further research is needed to examine whether individual counseling is effective among sedentary pregnant and postpartum women.

## Competing interests

The authors declare that they have no competing interests.

## Authors' contributions

MA participated in the design of the study, designed the protocol and contents of PA counseling, performed the statistical analysis and drafted the manuscript. MP participated in the design of the study, assisted with statistical analysis and helped to draft the manuscript. MF participated in designing the PA counseling and helped to draft the manuscript. TIK participated in the design of the study and helped to draft the manuscript. KO participated in the design of the study, planned the group exercise sessions and helped to draft the manuscript. RL conceived and coordinated the study and helped to draft the manuscript. All authors read and approved the final manuscript.

## Pre-publication history

The pre-publication history for this paper can be accessed here:



## Supplementary Material

Additional file 1 Table 1. Feasibility evaluation of the physical activity (PA) counseling procedure. Click here for file

Additional file 2 Table 2. Baseline information about the pregnant and postpartum participants in the two study groups according to baseline questionnaire and information obtained at the primary counseling visit. Click here for file

Additional file 3 Table 3. Weekly leisure time physical activity (LTPA) of the pregnant and postpartum participants in the experimental (EXP) and control group (CON) at baseline and two follow-ups, unadjusted arithmetic means (SD). Adjusted group differences (%), EXP compared with CON. Click here for file
