# Supplementary material for: Physical activity counseling in maternity and child health care – a controlled trial
Source: BMC Womens Health. 2008 Aug 14;8:14. doi: 10.1186/1472-6874-8-14 (PMC2527301; doi:10.1186/1472-6874-8-14)
Supplement: Additional file 1 — Table 1. Feasibility evaluation of the physical activity (PA) counseling procedure. [file 1472-6874-8-14-S1.doc]

Table 1. Feasibility evaluation of the physical activity (PA) counseling procedure.

| **Component and indicator** | **Evaluation method** |
| --- | --- |
| **Safety** |  |
| Incidence of selected adverse events^a)^ | Asked from all participants at the booster visits by the nurses. |
|  |  |
| Birth weight of the child | Obtained after delivery from maternity health cards of pregnant completers. |
|  |  |
| Average weeks’ gestation at the delivery | Obtained after delivery from maternity health cards of pregnant completers. |
|  |  |
| **Participant responsiveness** |  |
| Satisfaction with PA counseling | A 5-point Osgood scale (1=not at all satisfied … 5=very satisfied) in a questionnaire to all the participants. Returned in a sealed envelope to the nurse at the last follow-up. |
|  |  |
| Perceived effects of PA counseling on LTPA | A 6-point classification in a questionnaire to all the participants (1=no effects, 2=incentive to consider starting PA, 3=incentive to try out PA, 4=incentive to initiate PA, 5=incentive to maintain PA, 6=other effects). Returned in a sealed envelope to the nurse at the last follow-up. |
|  |  |
| **Realization of counseling** |  |
| Timing of the PA counseling sessions | Regarding each session a specific space was provided in the counseling card for weeks’ gestation and weeks from delivery. |
|  |  |
| Duration of the PA counseling sessions | Regarding each session a specific space was provided in the counseling card for the time counseling started and ended. |
|  |  |
| Number of PA counseling sessions missed | Nurses’ notes on the space provided for each session in the counseling card indicated that the session was completed, no notes indicated a missed session. |
| Adherence^b)^ to group exercise sessions | Participation lists of the group exercise instructors. |
|  |  |
| **Applicability** |  |
| Applicability of the primary PA counseling session to routine health care visits | A 5-point scale in a questionnaire returned anonymously by the nurses to the researchers after they had completed at least one primary session (1=inapplicable … 5=very applicable). |

^a)^ Vaginal bleeding, strong contractions (pregnant participants), dizziness, dyspnea, headache, chest pain, tiredness or fatigue, calf pain or swelling and musculoskeletal symptoms ― most classified as warning signs for exercise session termination by ACOG [13].

^b)^ Participation in ≥ 50% of the sessions available in EXP for each individual woman during their pregnancy.
