# Supplementary material for: Physical activity counseling in maternity and child health care – a controlled trial
Source: BMC Womens Health. 2008 Aug 14;8:14. doi: 10.1186/1472-6874-8-14 (PMC2527301; doi:10.1186/1472-6874-8-14)
Supplement: Additional file 2 — Table 2. Baseline information about the pregnant and postpartum participants in the two study groups according to baseline questionnaire and information obtained at the primary counseling visit. [file 1472-6874-8-14-S2.doc]

Table 2. Baseline information about the pregnant and postpartum participants in the two study groups according to baseline questionnaire and information obtained at the primary counseling visit.

|  | **Pregnant participants** | | **Postpartum participants** | |
| --- | --- | --- | --- | --- |
|  | EXP | CON | EXP | CON |
|  | **N=69** | **N=63** | **N=53** | **N=39** |
| Age, mean (SD) | 27.3 (5.2) | 28.5 (4.4) | 28.7 (4.2) | 28.1 (4.4) |
| BMI (kg/m^2^), mean (SD)^*)^ | 24.1 (3.9) | 22.7 (2.3) | 23.8 (3.9) | 22.9 (3.0) |
| BMI (kg/m^2^) ≥ 25 (%) | 31 | 18 | 31 | 16 |
| Non-smoking prior to pregnancy (%) | 66 | 80 | 68 | 60 |
| Polytechnic or academic degree (%) | 39 | 62 | 49 | 51 |

*) Based on self-reported height at the primary visit and weight measured by the nurse at the primary visit. Regarding pregnant participants, obtained only from the completers.
