# Supplementary material for: Physical activity counseling in maternity and child health care – a controlled trial
Source: BMC Womens Health. 2008 Aug 14;8:14. doi: 10.1186/1472-6874-8-14 (PMC2527301; doi:10.1186/1472-6874-8-14)
Supplement: Additional file 3 — Table 3. Weekly leisure time physical activity (LTPA) of the pregnant and postpartum participants in the experimental (EXP) and control group (CON) at baseline and two follow-ups, unadjusted arithmetic means (SD). Adjusted group differences (%), EXP compared with CON. [file 1472-6874-8-14-S3.doc]

Table 3. Weekly leisure time physical activity (LTPA) of the pregnant and postpartum participants in the experimental (EXP) and control group (CON) at baseline and two follow-ups, unadjusted arithmetic means (SD). Adjusted group differences (%), EXP compared with CON.

|  | Baseline | | I follow-up | | | | II follow-up | | | |
| --- | --- | --- | --- | --- | --- | --- | --- | --- | --- | --- |
|  | EXP | CON | EXP | CON | Group diff. (%)  EXP vs. CON^*)^ | 95% CI | EXP | CON | Group diff. (%)  EXP vs. CON^*)^ | 95% CI |
| **Pregnant participants** | Prior to pregnancy | | 16-18 weeks’ gestation | |  | | 37 weeks’ gestation | |  | |
|  | N=66-67^**)^ | N=62-63^**)^ | N=56-57^**)^ | N=57^**)^ | N=94-96^**)^ |  | N=40^**)^ | N=54-56^**)^ | N=85-89^**)^ |  |
|  |  |  |  |  |  |  |  |  |  | |
| • Number of days with at least moderate-intensity LTPA | 4.1 (2.2) | 4.6 (2.3) | 4.3 (2.0) | 4.0 (2.1) | -2 | -12 to 19 | 4.1 (2.0) | 2.9 (2.1) | 43 | 9 to 87 |
| • Minutes of at least moderate-intensity LTPA | 263 (222) | 242 (139) | 238 (151) | 201 (144) | 12 | -26 to 71 | 183 (101) | 131 (127) | 154 | 16 to 455 |
| • Number of days with light LTPA | 3.5 (2.4) | 3.7 (2.7) | 3.5 (2.3) | 3.8 (2.3) | -10 | -28 to 11 | 3.3 (2.2) | 4.7 (2.5) | -24 | -41 to -3 |
| • Minutes of light LTPA | 219 (294) | 174 (177) | 148 (140) | 170 (181) | -7 | -46 to 60 | 129 (120) | 264 (295) | -36 | -65 to 20 |
|  |  |  |  |  |  |  |  |  |  |  |
| **Postpartum participants** | Prior to pregnancy | | 5 months from delivery | |  | | 10 months from delivery | |  | |
|  | N=52-53^**)^ | N=37^**)^ | N=51-52^**)^ | N=37^**)^ | N=86-87^**)^ |  | N=45-46^**)^ | N=36-37^**)^ | N=78-81^**)^ |  |
|  |  |  |  |  |  |  |  |  |  | |
| • Number of days with at least moderate-intensity LTPA | 5.1 (2.9) | 4.7 (2.2) | 5.2 (1.9) | 4.9 (2.2) | 9 | -8 to 29 | 4.9 (2.2) | 5.1 (2.8) | 2 | -17 to 24 |
| • Minutes of at least moderate-intensity LTPA | 289 (195) | 260 (144) | 265 (148) | 290 (200) | 19 | -17 to 71 | 240 (140) | 265 (191) | 21 | -18 to 78 |
| • Number of days of light LTPA | 4.0 (2.4) | 4.5 (2.4) | 3.8 (2.4) | 4.5 (2.2) | -12 | -31 to 13 | 4.1 (2.4) | 4.7 (2.6) | -11 | -33 to 17 |
| • Minutes of light LTPA | 219 (204) | 232 (192) | 159 (123) | 248 (266) | -19 | -57 to 50 | 179 (148) | 247 (221) | -14 | -57 to 73 |

^*)^ Group differences at the end of follow-ups adjusted for baseline LTPA, age, BMI, smoking status and education as confounding factors in the analysis of covariance.

^**)^ Number of participants who had the information completed at the time of the follow-up.
